# A New Endoscopic Approach to Remove a Retained Stingray Barb

**DOI:** 10.7759/cureus.26990

**Published:** 2022-07-18

**Authors:** Joseph Palatchi Oldak, Juan Carlos Angulo-Lozano

**Affiliations:** 1 Orthopaedic Surgery, Hospital Angeles Universidad, Mexico City, MEX; 2 Orthopedics and Traumatology, Hospital Infantil Privado, Mexico City, MEX; 3 School of Medicine, Universidad Anahuac, Mexico City, MEX; 4 School of Medicine, Universidad Anahuac Mexico., Mexico City, MEX; 5 General Surgery/Urology, Hospital General de Mexico, Mexico City, MEX

**Keywords:** retained stingray barb, poisonous sea animals, retained foreign object, minimally access surgery, stingray injury

## Abstract

Stingray injuries usually happen when a person steps into one, resulting in the attack of the fish introducing a sting into the feet or leg in a defensive manner, causing significant pain and inflammation due to the venom. Retained stingray barb occurs in a low percentage of these accidents and may be difficult to diagnose, for this reason, it is always recommended to use imaging studies. In this case report, we present a 42-year-old man who presented to the orthopedic consult one week after a stingray injury with the diagnosis of retained stingray barb. The aim of this case report is to present a new surgical approach for stingray barb removal with a minimally invasive technique using arthroscopic equipment using the stingray barb entry site as the port to introduce the endoscopic tools.

## Introduction

Stingrays are a group of cartilaginous fish members of the Chondrichthyes class, the same as sharks and skates. These fish are also known as “rays” and can be equipped with one to three posterior barbs or blades that have rows of sharp spines and can easily perforate the skin [1]. There are more than 150 species of stingray all over the world and there are reported 750-3000 stingray injuries annually in the United States [2]. The stingrays are not known for being aggressive and attack in a defensive manner and tend to leave the area when they feel danger. Most of the injuries occur in males (80%) and in the lower extremities. The most common reason for the attack is stepping on the stingray [3]. 

Stingray can be divided into two: non-venomous and envenoming. Envenoming can cause pain and bleeding. Envenoming wounds are characterized by a combination of severe pain, bleeding, systemic reactions from the glandular tissues, and a delayed reaction to the retained foreign body [4]. Usually, there is significant bleeding, then the pain starts (15-90 min), and then the tissues surrounding the wound site begin to take on a reddish color that ends up becoming a blue-gray color [5]. 

Systemic manifestations that may be found in a stingray injury may include anxiety, diaphoresis, vomiting, diarrhea, headache, nausea, and hypotension. If the patient does not receive treatment, the pain could last several hours and could be extended to the entire extremity [6-7]. 

An X-ray should be taken to rule out a retained stingray barb. Magnetic resonance can be used to find hypointense, space-occupying foreign bodies retained in soft tissues [8]. Surgery should be consulted if it is necessary to repair the neuro-vascular bundle. In a retrospective study, only 1.6% of the cases of stingray attacks had a foreign body retained in the injury site. Most stingray injuries may be prevented by always observing the seafloors, especially in least-crowded beaches, and not intentionally provoking encounters with stingrays. Crossing bays and fresh water should always be undertaken with care, shuffling the feet in the bottom of the water.

The aim of this case report is to present a new surgical approach for removing a retained barb in a minimally invasive technique using arthroscopic equipment using the stingray barb entry site as the port to introduce the endoscopic tools. 

## Case presentation

**Figure 1:**
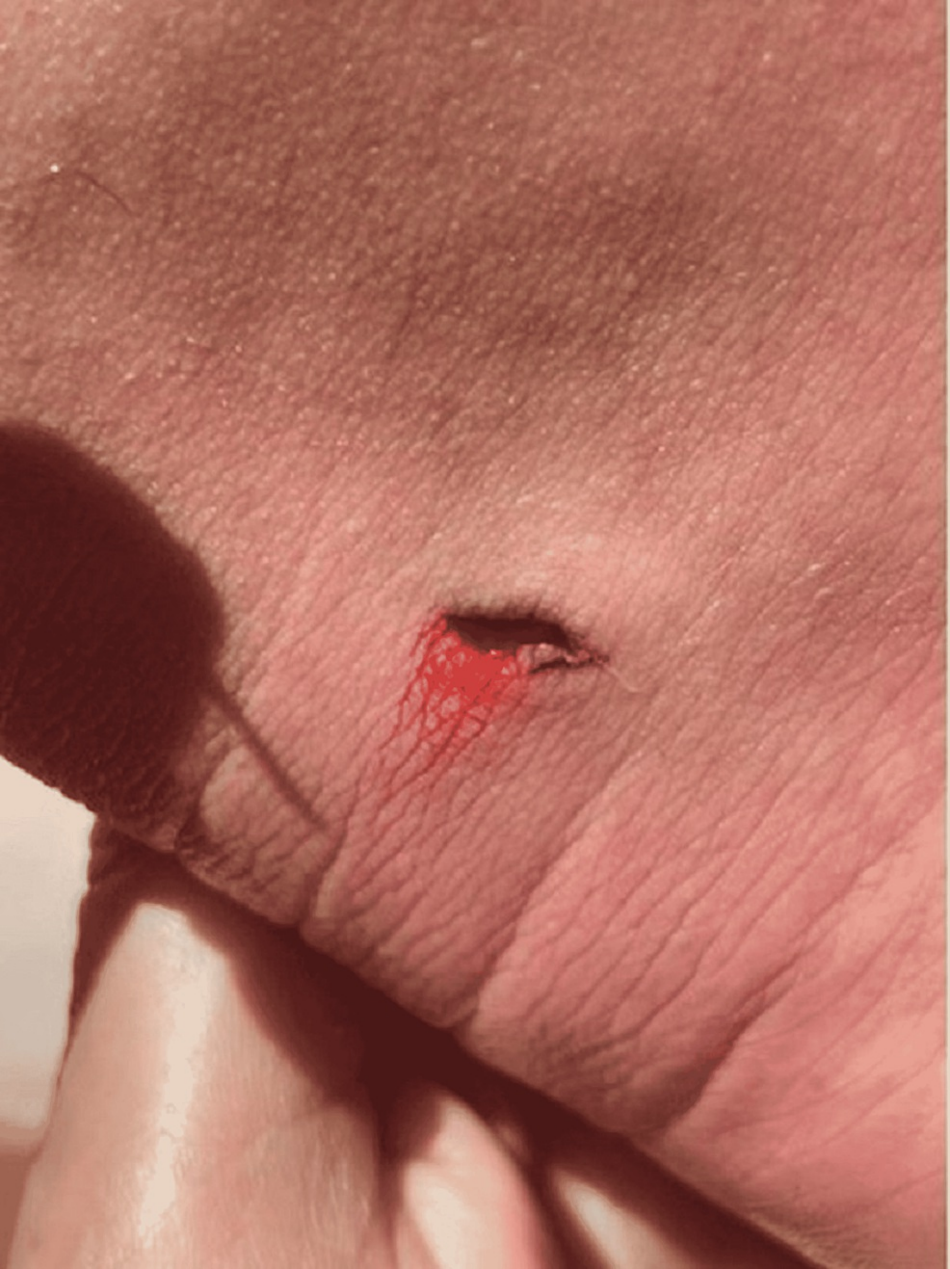
Stingray injury right after the accident, with no visible barb.

A previously healthy 42-year-old man was on vacation in La Paz, Baja California, Mexico. While walking along the coastline, the victim was stung by a stingray on his left ankle causing immediate intense pain and bleeding (Figure 1). 

He presented to a local emergency department, where the wound was cleaned with soap and water and he was prescribed Moxifloxacin 400 mg, Ketorolac 30 mg, and Dexketoprofen 25 mg. The wound was not explored, tetanus immunization was not provided, and no imagining was performed. 

On day 3 after the incident, the patient continued with pain, swelling, bleeding and difficulty to walk, so he went to a primary care physician who asked for laboratory tests where leukocytosis was found, prescribing Moxifloxacin 400 mg po every 24 h for 7 days, Ceftriaxone 1 g every 24 h for 3 days, Clindamycin 300 mg po every 8 h for 7 days and local wound care; again, no imaging was performed. 

**Figure 2:**
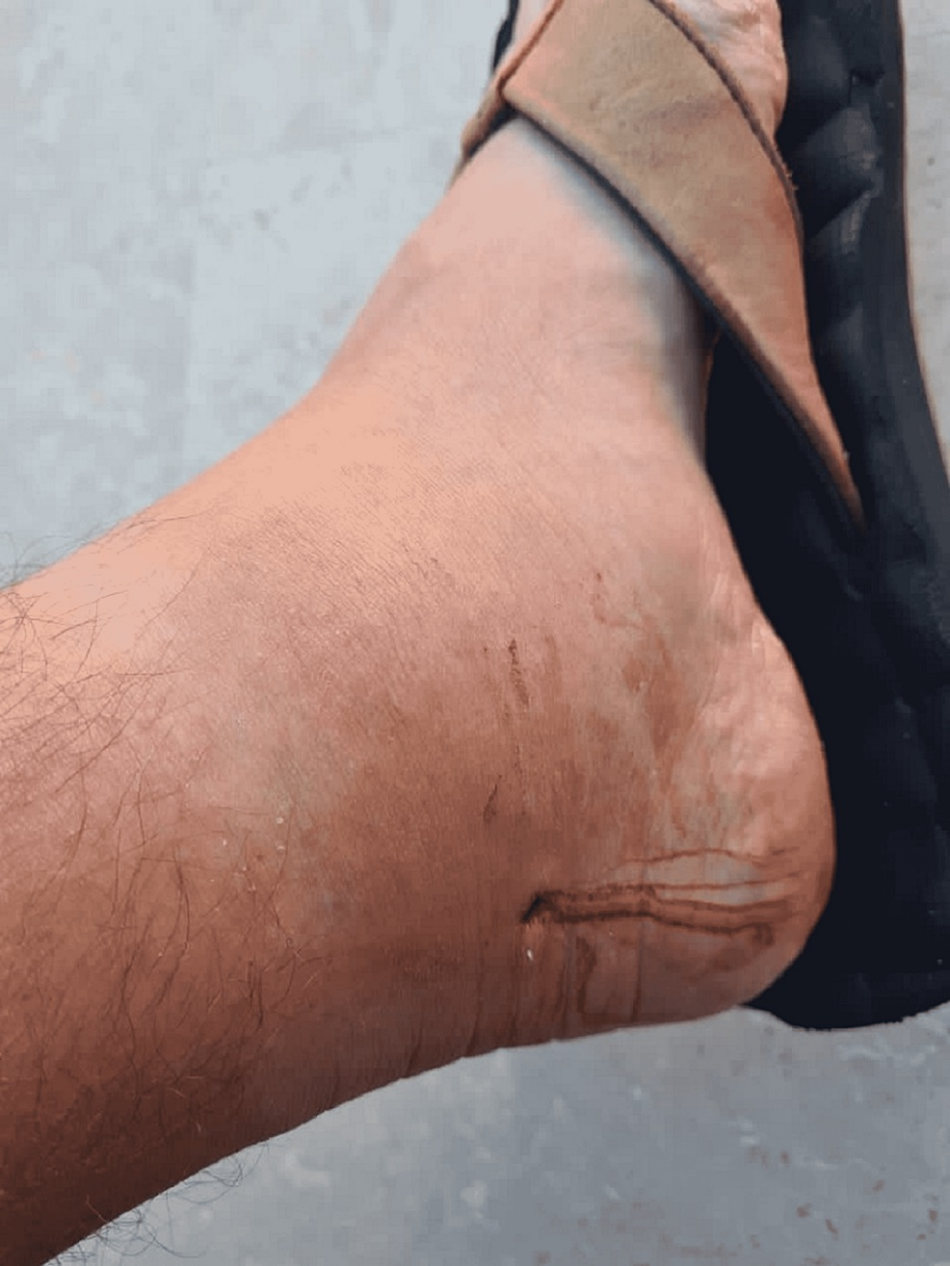
Foot after one week of the accident with serosanguineous crust draining from the open wound and perimaleolar inflammation.

One week after the initial injury, the patient returned to his home in Mexico City to have an appointment with his orthopedist as the swelling had progressed on his left ankle (Figure 2).

**Figure 3:**
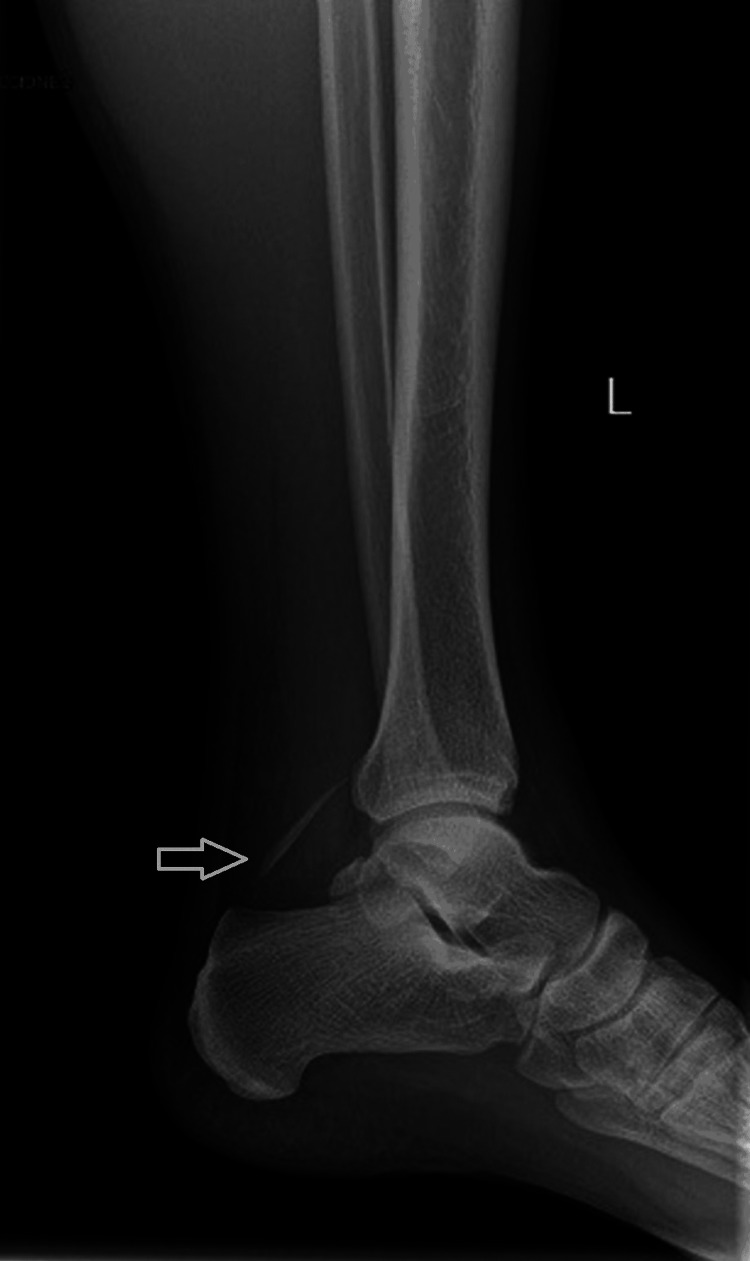
Lateral X-ray of the leg with a radiopaque object superior to the calcaneus marked by the arrow compatible with a stingray barb.

**Figure 4:**
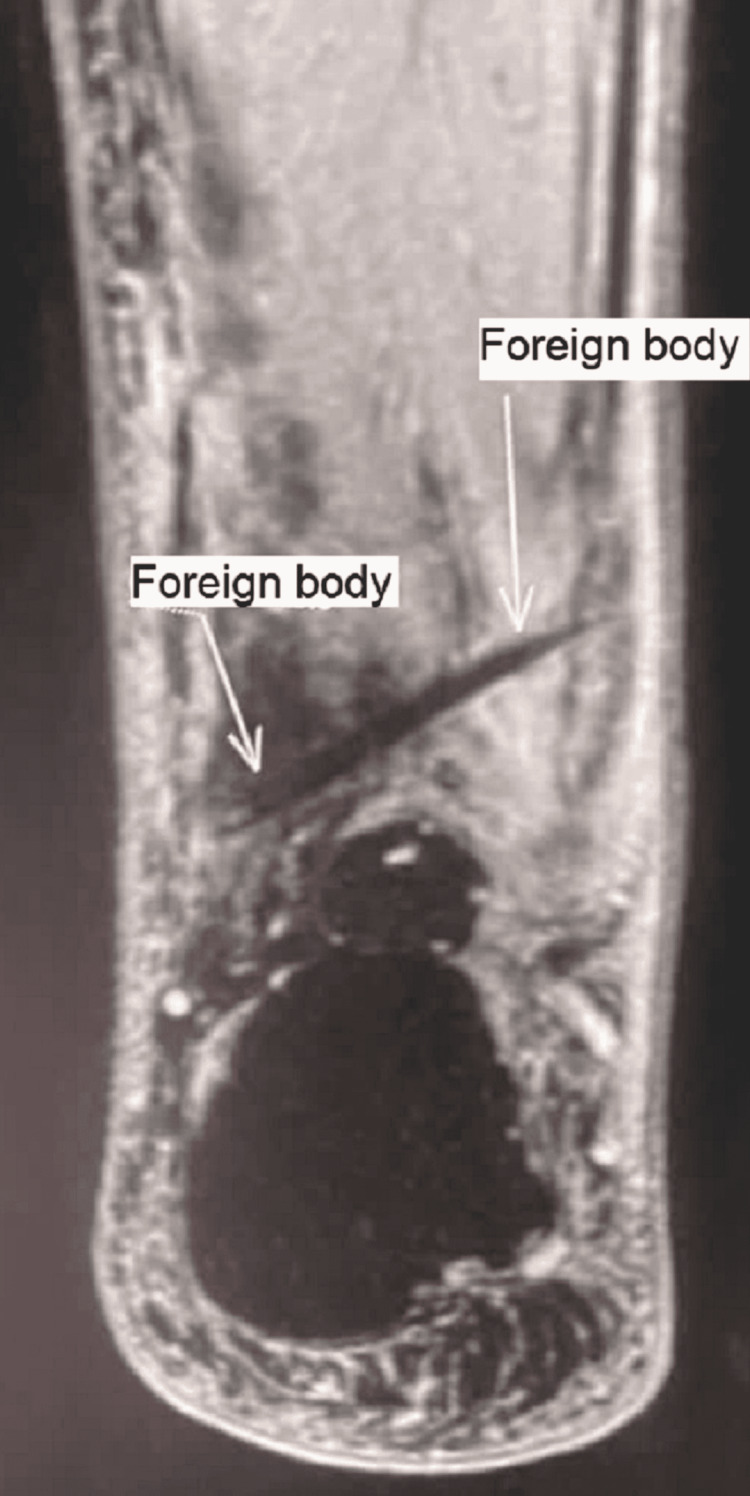
MRI in T2 sequence showing a foreign body marked by the arrows, hypointense, not compromising vascular or neural structures compatible with a barb.

The wound was still open, draining blood and purulent material. Sensibility was intact. An X-ray and MRI were performed, revealing a foreign body that had the appearance of a stingray spine (Figures 3-4). 

Bloodwork was ordered and the results of the complete blood count (CBC) biomarkers of inflammation were the following: 

**Table 1:** Bloodwork before surgery. ESR, erythrocyte sedimentation rate; CRP, C-reactive protein

Blood test	Result
Leukocytes	10,200/mm3
Neutrophiles	67%
Lymphocytes	25%
Hemoglobin	15.0 mg/dL
Hematocrit	44.7%
Platelets	212,000/mm3
ESR	3 mm/h
CRP	0.19 mg/dL

**Figure 5:**
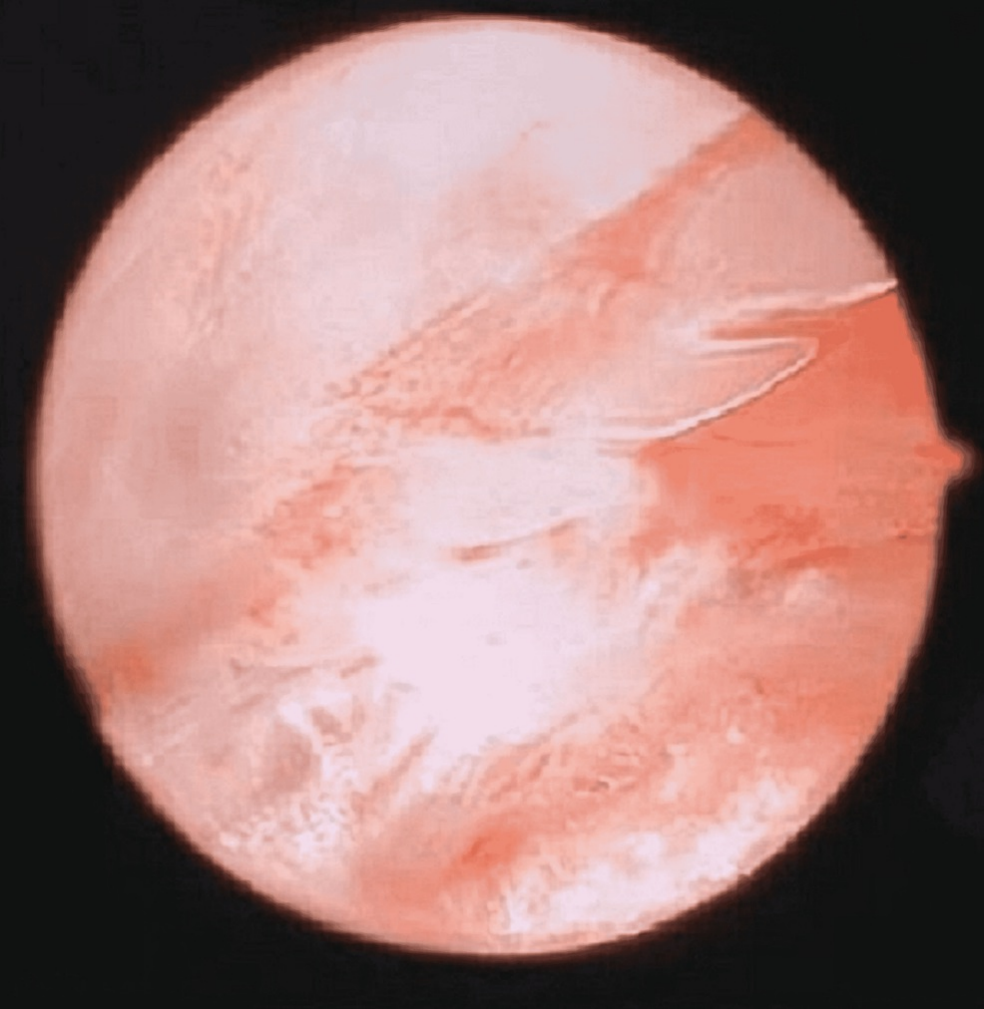
Endoscopic view of the stingray barb before its removal.

**Figure 6:**
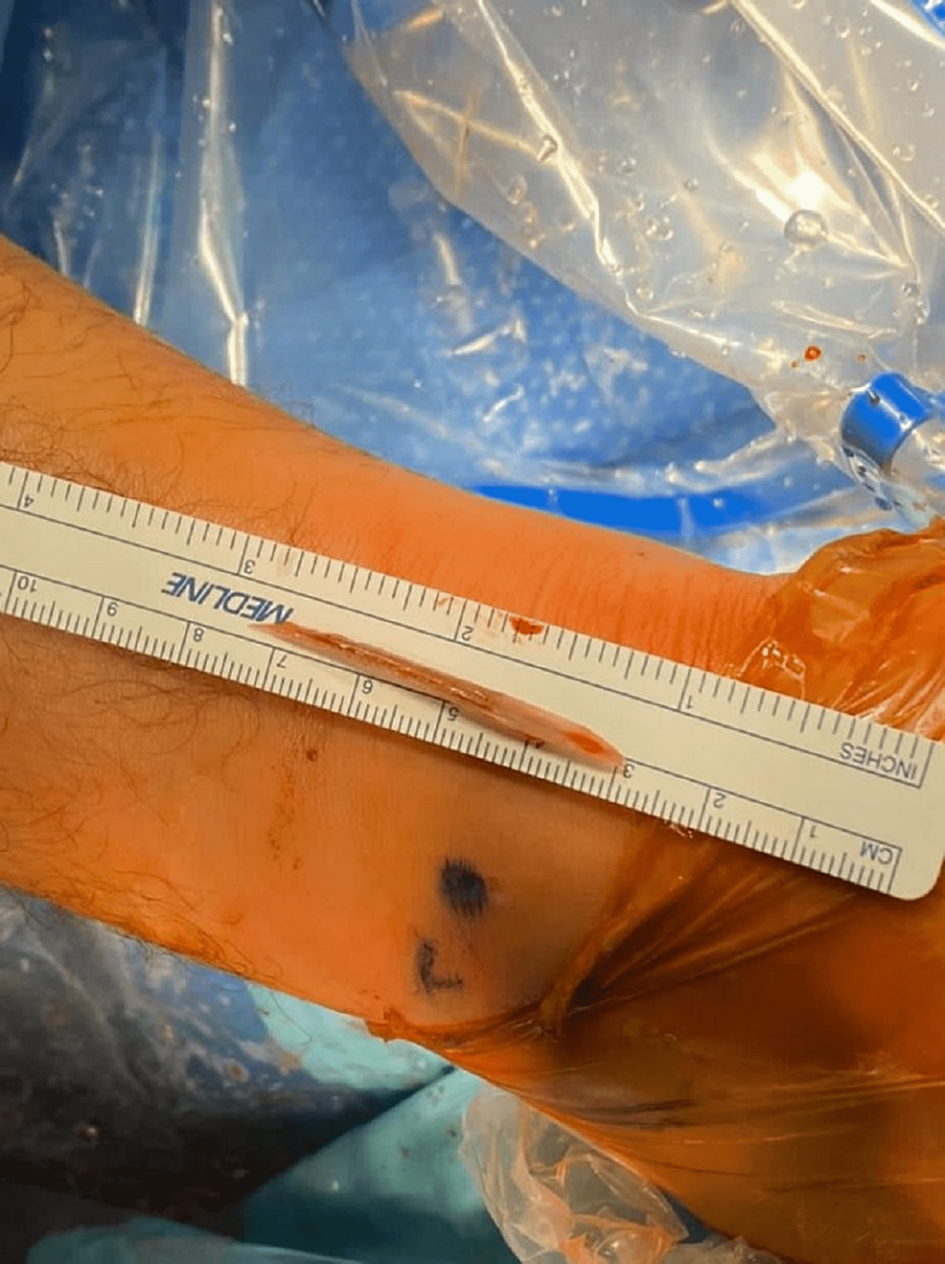
Stingray barb removed from the patient's ankle measuring 5.5 cm.

The patient was taken to the operating room, performing an endoscopic approach through the same entry hole of the stinger to avoid making a new contralateral wound (Figure 5). The stingray barb was removed with the arthroscopy equipment, dissecting surrounding tissue to avoid the laceration of the barb when pulling it out of the wound; the barb was successfully removed with no blood loss and preservation of the adjacent tissue (Figure 6).

He was sent home with the following medications: Cefixime 400 mg every 12 h for 7 days, Clindamycin 300 mg every 8 h for 7 days, Acetaminophen 750 mg every 8 h for 7 days, and Celecoxib 200 mg every 12 h for 5 days. After this, he was sent to physical rehabilitation. 

Follow-up one month after the surgery, the patient’s surgical incision was closed and well healed with no signs of inflammation or infection. He returned to his daily activities with no pain and with a normal gait. 

## Discussion

The stingray injuries should be managed as soon as possible, focusing on identifying the anatomic structures damaged, controlling the pain, performing imaging studies, and reducing the infection rates by washing properly the injury and giving empiric antibiotics [1-5]. 

The initial management should begin at the scene and continue at the nearest hospital. Seawater can be used to remove fragments of the spine, glandular fragments of the spine, and glandular tissue. Any significant bleeding should be staunched with local pressure only. If available, the wound should be cleaned with hot tolerable water to inactivate the heat-liable venom [3, 7]. On arrival at the hospital, appropriate analgesia should be established, using parenteral analgesics, and tetanus prophylaxis should be administered. In case of a lot of pain, regional nerve blocks without epinephrine can be used [1]. 

In this case, a new treatment technique is described for the approach of a retained stingray barb, in which an endoscopic approach is performed through the same stinger entry hole instead of performing a new contralateral wound. With this new technique described, it is possible to reduce the risk of infection by having just one incision, tissue injury, or damaging the neurovascular bundle, improving post-surgical pain, and having a faster recovery [2, 8].

Evaluation of nearby important anatomic structures is fundamental when approaching the barb. Once the foreign body is identified it is recommended to expand the wound with the forceps for a better view and smoother extraction. Debridement of any tissue that is stuck with the barb that could impede its removal should be cleaned. The exterior barb tip should be secured and extracted in a parallel manner to the canal. Arthroscopic exploration should be made after its extraction for possible barb debris and washing with saline solution is recommended. 

Prophylactic antibiotic therapy should be started at the emergency department, and directed at common marine bacteria including streptococcal, staphylococcal, and Vibrio species. Although evidence on the efficacy of prophylactic antibiotics is limited, there is a higher rate of visits to the emergency department with symptoms suggesting wound infection among patients who did not receive antibiotics [5]. 

In a retrospective study of 119 patients with stingray injury, only 58% of the patients had imaging of the injury site [2]. This case is an example of the importance of imaging when encountering a stingray injury as the barb may not be visible on physical examination and recovery may be delayed. For this reason, we suggest the use of imaging in all patients with a stingray injury. Retained stingray barb may present as a painful injury with no healing, purulent secretion in the injury site, erythema, low-grade fevers, and limp as in this case [9]. 

## Conclusions

Most stingray injuries are not fatal and heal without complications. To avoid a delay in the diagnosis and treatment of a retained stingray barb, it is necessary to be very precise in the physical examination and perform imaging studies (X-Ray, CT scan, or MRI). In this case report, we successfully removed a stingray barb using the wound as the entry site with arthroscopic equipment for minimally invasive surgery and faster recovery.

## References

[1] Diaz JH (2008). The evaluation, management, and prevention of stingray injuries in travelers. J Travel Med.

[2] Clark RF, Girard RH, Rao D, Ly BT, Davis DP (2007). Stingray envenomation: a retrospective review of clinical presentation and treatment in 119 cases. J Emerg Med.

[3] O'Connell C, Myatt T, Clark RF, Coffey C, Nguyen BJ (2019). Stingray envenomation. J Emerg Med.

[4] Tartar D, Limova M, North J (2013). Clinical and histopathologic findings in cutaneous sting ray wounds: a case report. Dermatol Online J.

[5] Falk DP, Metikala S, Lopez VS, Stein M, Mahmoud K, Chao W (2019). Late presentation of a retained stingray spine in the plantar medial hindfoot. Foot Ankle Orthop.

[6] Bendt RR, Auerbach PS (1991). Foreign body reaction following stingray envenomation. J Wilderness Med.

[7] Hønge BL, Patsche CB, Jensen MM, Schaltz-Buchholzer F, Baad-Hansen T, Wejse C (2018). Case report: iatrogenic infection from traditional treatment of stingray envenomation. Am J Trop Med Hyg.

[8] Srinivasan S, Bosco JI, Lohan R (2013). Marine stingray injuries to the extremities: series of three cases with emphasis on imaging. J Postgrad Med.

[9] O'Malley GF, O'Malley RN, Pham O, Randolph F (2015). Retained stingray barb and the importance of imaging. Wilderness Environ Med.

